# Multiphase Processing of the Water-Soluble and Insoluble Phases of Biomass Burning Organic Aerosol

**DOI:** 10.1021/acsestair.4c00345

**Published:** 2025-03-31

**Authors:** Habeeb H. Al-Mashala, Meredith Schervish, Sithumi M. Liyanage, Jace A. Barton, Manabu Shiraiwa, Elijah G. Schnitzler

**Affiliations:** † Department of Chemistry, Oklahoma State University, Stillwater, Oklahoma 74078, United States; ‡ Department of Chemistry, University of California Irvine, Irvine, California 92697, United States

**Keywords:** Reactive uptake, atmospheric aging, brown carbon, ozone, relative humidity, sunlight

## Abstract

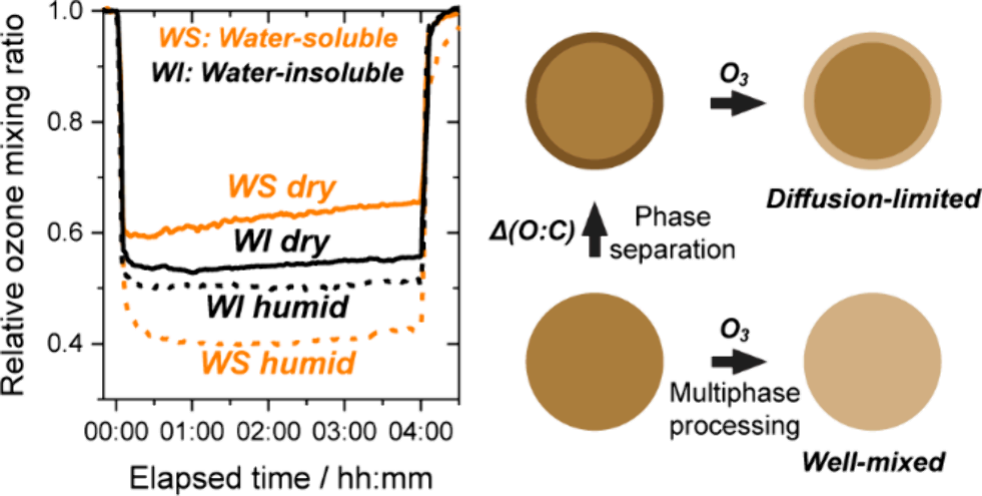

Biomass burning is one of the most significant sources of organic aerosol in the atmosphere. Biomass burning organic aerosol (BBOA) has been observed to undergo liquid–liquid phase separation (LLPS) to give core–shell morphology with the hydrophobic phase encapsulating the hydrophilic phase, potentially impacting the evolution of light-absorbing components, i.e., brown carbon (BrC), through multiphase processes. Here, we demonstrate how multiphase processing differs between the water-soluble (i.e., hydrophilic) and insoluble (i.e., hydrophobic) phases of BBOA in terms of reactive uptake of ozone in a coated-wall flow tube. Effects of relative humidity (RH) and ultraviolet (UV) irradiation were investigated. Experimental timeseries were used to inform simulations using multilayer kinetic modeling. Among non-irradiated thin films, the uptake coefficient was greatest for the water-soluble phase at 75% RH (3 × 10^–5^, corresponding to a diffusion coefficient of BrC, *D*_BrC_, of 3 × 10^–9^ cm^2^ s^–1^) and least for the same phase at 0% RH (1 × 10^–5^, corresponding to *D*_BrC_ of 1 × 10^–10^ cm^2^ s^–1^). The uptake coefficient for the water-insoluble phase fell between these two (about 1.5 × 10^–5^), regardless of RH, and the corresponding *D*_BrC_ increased only slightly (8 × 10^–10^ cm^2^ s^–1^ at 0% RH to 9 × 10^–10^ cm^2^ s^–1^ at 75% RH). The uptake coefficients of both phases at 0% RH decreased significantly after UV irradiation, consistent with a transition from viscous liquid to solid and supported by qualitative microscopy observations. Modeling multiphase ozone oxidation of primary BrC components in the atmosphere demonstrated, first, that LLPS may extend the lifetime of water-soluble BBOA encapsulated by water-insoluble species by a factor of 1.5 at moderate to high RH and, also, that UV irradiation may extend the lifetime of both phases by more than a factor of 2.5.

## Abstract 2

Liquid−liquid phase separation and ultraviolet irradiation may restrict the multiphase processing of biomass burning organic aerosol governing its climate effects.

## Introduction

Biomass burning is one of the most significant sources of organic aerosol in the atmosphere.^1^ Biomass burning organic aerosol (BBOA) is a complex mixture of molecules with many oxygenated functional groups, including a wide range of phenolic species from the thermal degradation of lignin.^2,3^ These species have diverse physical, chemical, and optical properties.^4^ Components of brown carbon (BrC) effectively absorb visible light, so they impact climate directly by absorbing in addition to scattering solar radiation.^5,6^ Many compounds are also polar, so they take up water, such that BBOA particles can grow to sizes at which they can act as cloud condensation nuclei. BBOA can undergo physical and chemical aging during its one-to-two-week residence time in the troposphere. As BBOA travels downwind from its source, the most volatile components evaporate as the plume is diluted,^7,8^ resulting in changes to the volatility distribution, as well as light absorption and viscosity.^9−11^ As it travels downwind or aloft, BBOA is also exposed to oxidants, including ozone,^12,13^ hydroxyl radical,^14,15^ and nitrate radical,^16,17^ which can drive multiphase processing that also changes its light absorption.^18^ Ozone, specifically, has a whitening effect on BrC in BBOA.^12,13^ This multiphase processing depends on chemical kinetics, i.e., rate constants, as well as physical properties, i.e., viscosity.^19,20^ If viscosity is high, multiphase processing is inhibited as diffusion of ozone into the particle bulk is slowed and replenishment of BrC components to the surface is also kinetically limited.^13,21^

One of the properties that is highly variable among BBOA components is polarity.^22,23^ For internal mixtures of organic aerosol components of two different origins, large differences in polarity, quantified in terms of oxygen-to-carbon ratio (O:C), have been shown to lead to liquid–liquid phase separation (LLPS), with a threshold Δ(O:C) of 0.3–0.5.^24,25^ Recently, LLPS has been observed specifically in BBOA, resulting in core–shell particles with a hydrophilic core and a hydrophobic shell.^21^ Through direct measurements of bulk diffusion coefficients, the viscosities of these phases and their responses to relative humidity (RH) have been shown to differ.^21^ For example, the hydrophilic phase was more viscous than the hydrophobic phase at low RH, but the hydrophobic phase was more viscous at moderate to high RH. These viscosities suggest that, at the moderate to high RH values common in the atmosphere, the hydrophobic shell can limit diffusion of oxidants to the core and consequently prolong the lifetime of BrC in the atmosphere with respect to multiphase processing;^21^ e.g., the shell could prevent ozone from reaching the core. However, there is a lack of direct measurements of the multiphase processing of the separate hydrophilic (i.e., water-soluble) and hydrophobic (i.e., water-insoluble) phases of BBOA.

Here, our major focus is the hypothesis that liquid–liquid phase separation will influence the lifetime of BBOA due to distinct changes in multiphase processing of the hydrophilic and hydrophobic phases with changes in RH. Consequently, we investigate the multiphase processing of the two phases at low and high RH. Our minor focus is the hypothesis that irradiation of separate hydrophobic and hydrophilic phases will lead to increased viscosity, an effect previously observed only for whole BBOA containing both polarity fractions.^26^ Consequently, we also investigate the multiphase processing of the two phases at low RH with and without irradiation. BBOA was generated through thermal degradation of biomass in the laboratory and separated by polarity through extractions of filter-collected samples in water and methanol, sequentially. These extracts were used to prepare thin films, which were exposed to ozone in a coated-wall flow tube. We applied multiphase kinetics modeling to reproduce the measured ozone uptake coefficient, estimating the diffusion coefficients of ozone and BrC in the two phases of BBOA under different conditions. Finally, the model was further applied to make estimates of the atmospheric lifetime of BrC in the two phases of BBOA with respect to multiphase ozone oxidation.

## Materials and Methods

### BBOA Generation

BBOA was generated from the sapwood of eastern red cedar, following the procedure described in detail earlier.^26^ Eastern red cedar was chosen because it is a representative fuel species for biomass burning in the Southern Great Plains, a region where the incidences of severe wildfires have increased in number significantly between 1984 and 2011.^27^ In this region, eastern red cedar is a native woody species encroaching on tallgrass prairie; in Oklahoma, specifically, its land coverage area increased by 8% annually between 1984 and 2010.^28,29^ This encroachment increases the fuel loading and risk of wildfire,^29,30^ so eastern red cedar is also targeted in prescribed burns in the region.^30−32^ Sapwood was chosen because it may be consumed in both low- and high-temperature fires, unlike heartwood, and accounts for a significant mass fraction of fuel, unlike bark.

Briefly, a rectangular chip of sapwood was placed in a 5 cm diameter quartz flow tube, held in a tube furnace (Across International, STF1200) in a fume hood. Optimal operating conditions for thermal degradation without flaming included a flow of 2 L min^–1^ of ultrapure air (Matheson, Zero Gas) through the tube and a 15 min linear ramp in temperature from room temperature to 673 K. Thermal degradation began at 653 K, at which point a 47 mm diameter filter (Pall, Emfab) was placed in line to collect the BBOA particles. Filter-collected samples of about 100 mg were stored in a freezer at approximately 260 K between generation and use.^33^ The mass and mass fractions of the hydrophilic and hydrophobic phases were consistent across experiments, as described in our discussion. All masses were measured gravimetrically on an analytical balance.

### Thin Film Preparation

Filter-collected BBOA was used for the preparation of thin films, mostly following the procedure described earlier,^26^ except for the important difference of separating water-soluble and insoluble components. First, filters with BBOA were placed in line with 3 L min^–1^ of clean, dry air from a commercial zero-air generator (Aadco, 747–30) for 48 h to remove the most volatile BBOA components that might otherwise interfere with the later reactive uptake measurements by reacting with ozone in the gas phase.^10,34^ Water-soluble components were extracted in 10 mL of ultrapure water (18 MΩ cm) in a vial on a roller mixer for 15 min. The resulting aqueous solution was placed on a rotary evaporator at about 6 kPa and 313 K for 15–20 min, until dry, and quantitatively transferred to a vial in methanol. The methanol was then evaporated under nitrogen (Matheson, Ultra High Purity), and the extract was stored in the freezer. After extraction of the water-soluble components, the filter was removed from the vial and dried with nitrogen. The remaining water-insoluble components were extracted from the filter in 8 mL of methanol (Fisher, > 99.8%). The resulting solution was passed through 0.45-μm syringe filter, blown down with nitrogen at 3 L min^–1^, and stored in the freezer.

To prepare a thin film from the water-soluble or insoluble components, methanol was added to the extract to give a mass concentration of 10 mg mL^–1^. All volumes were dispensed using calibrated pipettors. For water-insoluble components, which make up a smaller fraction of the BBOA mass, extracts from multiple filters had to be combined. A 4 mL aliquot of this solution was then added to a borosilicate glass tube with machined, sealed spacers that control the length of the thin film. The glass tube containing solution was slowly rotated on a level roller mixer, with clean, filtered air flowing through it, over the solution, to speed the evaporation of methanol. The resulting thin films were translucent and uniform in color.

### UV Irradiation

Some thin films were exposed to UV irradiation in a custom-built cylindrical photoreactor, described in detail earlier.^26,35^ Briefly, a glass tube supporting a thin film was placed vertically at the radial center of the photoreactor and irradiated with 16 UV–B bulbs (Ushio, G8T5E) with peak emission at 310 nm for 16 h. Previously, chemical actinometry was performed for borosilicate glass, using *p*-nitroanisole/pyridine,^36−39^ to show that this exposure is approximately equivalent to 3 d in the atmosphere; i.e., 3 d suspended, since we compare to the 24-h average solar spectral flux.^26^ The temperature in the photoreactor during irradiation was 308 K.

### Spectroscopic Analysis

The absorbance of the water-soluble and insoluble fractions used to prepare the thin films with and without UV irradiation was measured in methanol, at known mass concentrations, using a modular fiber-optic-based spectrometer (Ocean Insight, Flame-T-UV–vis).^35,40^ The spectrometer is fitted with a balanced deuterium halogen light source (DH-2000-BAL) and a 1 cm cuvette holder with lenses (Thorlabs, CVH100).

### Reactive Uptake

**Figure 1:**
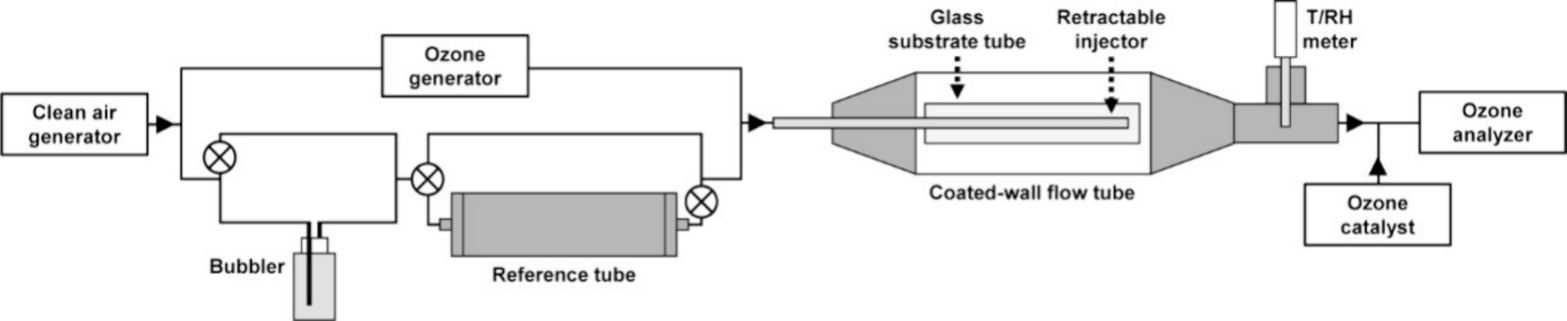
Coated-wall flow-tube setup. The reference tube housed the glass substrate tube and thin film while testing for volatile components reacting with ozone in the gas phase, and it was empty and bypassed during reactive uptake of ozone onto the thin film.

Reactive uptake of ozone onto the thin films of water-soluble and insoluble BBOA was measured using a coated-wall flow tube with a retractable injector, as shown in Figure 1, which has been described in detail previously.^26,41^ The sample was first placed in a reference tube upstream of the introduction of ozone; the rationale for this step was to test for potential gas-phase consumption of ozone by any reactive semivolatile components remaining after BBOA treatment. For films prepared from the water-soluble phase, there was a 4 ± 1% loss of ozone, while for those prepared from the water-insoluble phase, there was no loss; this slight difference between the phases suggests that the water-insoluble phase contained a smaller fraction of low molecular-weight species after treatment than the water-soluble phase. The sample was then placed in the flow-tube, and the injector was retracted to condition the film at 0% or 75% RH for 30 min. Once the film was conditioned, the injector was pushed forward, and ozone generation began. After a steady initial mixing ratio was obtained, the moveable injector was retracted 5 cm for 4 h, before being returned to its initial position. The initial mixing ratio of ozone, generated using a Hg lamp (UVP, SOG-2), was set to about 130 ppb, measured using a UV absorption analyzer (Ecotech, Serinus 10). RH was monitored by a temperature and RH probe (Vaisala, HMP75).

The uptake coefficient, the ratio of reactive to total collisions, was determined from the loss of ozone, as described previously.^26,41^ First, the experimental rate constant, *k*_obs_, was determined according to eq 1:
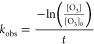
1where [O_3_]_0_ is the initial ozone mixing ratio without retracting the injector, [O_3_] is the current ozone mixing ratio, and *t* is the residence time of ozone in the glass substrate tube. Next, the effective uptake coefficient, γ_eff_, was determined according to eq 2:
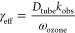
2where *D*_tube_ is the inside diameter of the substrate tube, and ω_ozone_ is the mean speed of ozone in the gas phase. Last, the corrected uptake coefficient, γ_corr_, was determined according to eq 3:
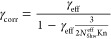
3where *N*_Shw_^eff^ is the Sherwood number, and Kn is the Knudsen number.^42,43^ Physical parameters of the setup are presented in Table S1.

### Microscopy

Qualitative microscopy experiments were performed to determine whether thin films exhibited flow or not, consistent with a viscous liquid or solid, respectively.^44^ For these experiments, thin films were prepared on flat circular glass coverslips (VWR, 48382–042), rather than the glass tubes used for uptake experiments. A 120-μL aliquot of an extract of water-insoluble BBOA in methanol, at a mass concentration of 10 mg mL^–1^, was dispensed on a coverslip, and the methanol was allowed to evaporate. The thin films were investigated at 35% RH and 298 K on a trinocular compound microscope (AmScope, CL-T490B). Initially, a thin film was scraped with the square edge of the end of a precision cutting knife blade. The initial appearance and evolution of the resulting groove in the thin film were determined 0, 2, and 20 h after scraping.

### Kinetic Multilayer Modeling

The kinetic multilayer model of gas-particle interactions in aerosols and clouds (KM-GAP) was used to simulate the flow tube conditions and to reproduce the measured uptake coefficients of ozone to the thin films of water-soluble and water-insoluble biomass burning material under different RH conditions. KM-GAP was then used to simulate the lifetime of BrC components in atmospheric aerosol particles exposed to atmospherically relevant ozone mixing ratios. The details of KM-GAP and of its application to flow tube experiments have been described previously,^45−47^ but a brief description is given here, as well as the specifics of this implementation.

KM-GAP consists of a gas phase, a near-surface gas phase, a sorption layer, a near-surface bulk layer, and a number of bulk layers. KM-GAP explicitly treats the processes of gas-phase diffusion, adsorption and desorption, surface-to-bulk transport, and bulk diffusion within the thin film or particle. The reaction between ozone and BrC is simulated in the near-surface bulk layer and all the bulk layers of the film and aerosol particles. The BrC and the oxidation product were assumed to be nonvolatile and have molecular weights of 248 g mol^–1^ and densities of 1.3 g cm^–3^.^3,48^ The Henry’s law constant for ozone in the biomass burning material film was set at 0.24 M atm^–1^ as in Gregson et al.^21^ This value is chosen based on experimental data to be in between the Henry’s law constant of ozone in oleic acid and water, as a value for biomass burning organic material is unknown.

For the simulated flow tube setup, the number of layers in the film bulk was set to 100, and a flat geometry was implemented by using a constant surface area for each layer. As in the experimental setup, the film was exposed to 130 ppb of ozone, which was constantly replenished at the experimental flow rate. The ozone uptake coefficient was calculated as the difference between the adsorption and desorption flux divided by the collision flux. The ozone and BrC bulk diffusivities in the film, the reaction rate coefficient between ozone and BrC, and the initial concentration of reactive BrC were chosen from the best fit to the experimental uptake coefficient data. The diffusivities were allowed to vary in all experiments: with RH, between the irradiated and nonirradiated cases, and between the water-soluble and water-insoluble biomass burning films. The reaction rate coefficient was fixed to be the same for all experiments. The initial concentration of BrC was only varied between the nonirradiated and irradiated cases, but fixed to be the same for all water-soluble and water-insoluble biomass burning films across the RH range. While parameters may be different under the scenarios, they are fixed; here, there are no experimental constraints for these parameters. Therefore, this set of fixed parameters was chosen to minimize the parameters to optimize in order to achieve a unique solution, where the main tunable parameters across the experiments were the diffusivities.

To calculate BrC lifetimes in atmospheric aerosol particles, particles with diameter of 300 nm were exposed to atmospherically relevant mixing ratios of ozone ranging from 10 to 50 ppb. The number of layers in the particle bulk was set to be 10. The best fit values for the rate coefficient between ozone and BrC, the diffusivities of ozone and BrC in the biomass burning material, and the initial concentration of BrC in each experiment were used to simulate the decay of BrC due to reaction with ozone in the particle. The lifetime of BrC was calculated as the e-folding time scale.

## Results and Discussion

### Characteristics of Polarity Fractions

BBOA was generated in the laboratory from the sapwood of eastern red cedar. In all, 28 thermal decomposition experiments were performed to generate BBOA. The evaporation of the most volatile components after passing clean air through the filter-collected samples led to about a 45% decrease in BBOA mass.^26^ This evaporation is necessary experimentally to attribute the loss of ozone solely to multiphase chemistry,^10,34^ and it also mimics the evaporative aging of BBOA immediately downwind of the source due to dilution.^11^ Furthermore, the remaining material may better represent ambient BBOA, which has been shown to be more viscous than nascent laboratory-generated BBOA.^49^ Afterward, the average total mass of BBOA remaining on a filter was 51 ± 7 mg. Of this, 40 ± 8 mg was extractable in water, and 11 ± 3 mg remained on the filter. The percentage of BBOA that is water-soluble, i.e., about 78%, is in good agreement with previously reported values for BBOA from pine and cedar, both roughly 75%.^50^ The remaining water-insoluble mass was extracted quantitatively into methanol, in agreement with previous extractions of 99% of the whole BBOA mass, without separation of water-soluble components, into methanol.^26^ In other words, if the order of extraction were reversed, negligible BBOA material would remain after methanol. The generation of BBOA was performed under conditions where there was no flaming; under other conditions, a significant fraction of methanol-insoluble material can be generated.^51−53^ These extracts were used to prepare thin films with thickness of about 6 μm. Before each thin film was used for a reactive uptake experiment, it was verified that consumption of ozone through gas-phase reaction was negligible, so all losses below can be attributed to multiphase reaction.

The initial absorptivities of the water-soluble and insoluble phases were characterized in terms of the mass absorption coefficient (MAC), i.e., the absorbance normalized to the mass concentration.^4^ We note that the MAC values after the preparatory evaporation performed here are higher than those of fresh BBOA, since the most volatile components are least absorbing.^11,26^ The MAC values for the water-soluble phase of BBOA at 365 and 405 nm were 0.51 ± 0.06 m^2^ g^–1^ and 0.15 ± 0.03 m^2^ g^–1^, respectively, calculated from the spectra in replicate experiments shown in Figure S1. These yield an absorption Ångström exponent, AAE, of 12, a reflection of the strong wavelength dependence of absorbance. The MAC value at 365 nm, 0.51 m^2^ g^–1^, lies within the range observed for water-soluble organic carbon in the field, 0.2–1.1 m^2^ g^–1^.^4,54^ The MAC values for the water-insoluble phase at 365 and 405 nm were 1.20 ± 0.03 m^2^ g^–1^ and 0.42 ± 0.04 m^2^ g^–1^, each more than twice that of the water-soluble phase at the respective wavelength, and the resulting AAE is 10, the same as that of whole BBOA prepared similarly.^26^ The mass-weighted average of the two MAC values at a given wavelength is that of the whole BBOA; e.g., 0.51 m^2^ g^–1^ scaled by 0.78 added to 1.20 m^2^ g^–1^ scaled by 0.22 gives 0.66 m^2^ g^–1^, in good agreement with the previously reported value of 0.70 m^2^ g^–1^.^26^ In other words, since the water-soluble phase contributes most of the mass, the MAC value of the whole BBOA is closest to that of the water-soluble phase, although the two phases contribute similarly to the total absorption.

### Effects of Polarity and Relative Humidity

**Figure 2:**
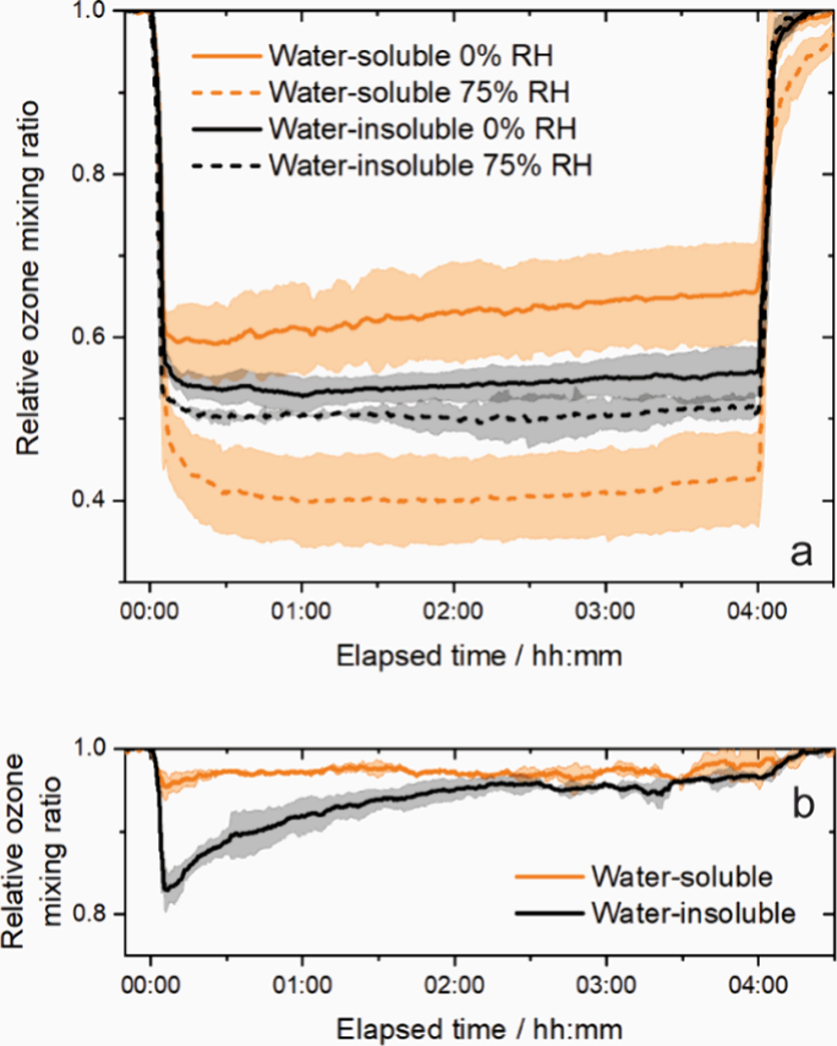
Relative ozone mixing ratio as a function of elapsed time for (a) nonirradiated and (b) irradiated BBOA. In the bottom panel, the RH is 0%. Each timeseries is the average of triplicate experiments, and the shaded region about each shows the variance between triplicates in terms of one standard deviation. In all, 18 uptake experiments were performed.

**Figure 3:**
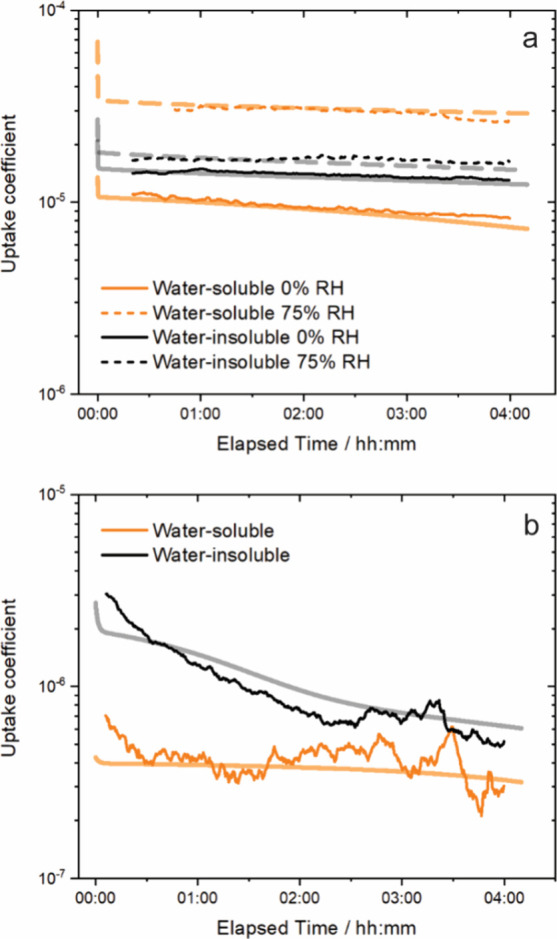
Experimental and simulated uptake coefficients as a function of elapsed time for (a) nonirradiated and (b) irradiated BBOA. In the bottom panel, the RH is 0%. Each timeseries is the average of triplicate experiments. The variance between triplicates in terms of one standard deviation is omitted here for clarity of presentation but shown in Figure S2.

We first investigated reactive uptake of ozone onto thin films of the water-soluble phase of BBOA. Previously, we investigated reactive uptake of ozone onto whole BBOA, including both water-soluble and insoluble components, at 0% RH.^26^ For pine BBOA, Gregson et al. showed that on a figure of diffusion coefficients versus RH, an intersection occurs at 35–40%, below which the hydrophilic phase is more viscous and above which the hydrophobic phase is more viscous.^21^ For this study, we chose 0 and 75% RH, which we call low and high RH, as these values are roughly equal increments from this intersection. For water-soluble BBOA at 0% RH, the ozone mixing ratio dropped initially by 40%, as shown in Figure 2a. This loss of ozone is associated with an uptake coefficient of about 1 × 10^–5^, as shown in Figure 3a. No mass loss of the thin film was observed after ozone exposure; since volatilization does not occur, all consumption of ozone throughout the experiments is due to multiphase reactions. After a 4-h exposure, the ozone mixing ratio increased slightly, such that the loss was about 35% of the initial mixing ratio. This extent of reactive uptake is less than that previously observed for the mixed water-soluble and insoluble components; i.e., for whole BBOA, similarly generated from the sapwood of eastern red cedar, the relative ozone mixing ratio initially dropped more than 50%, and the uptake coefficient was almost 1.5 × 10^–5^.^26^ At 75% RH, the ozone mixing ratio decreased more significantly, by 60%, and this was mostly steady for the duration of the experiment. The associated uptake coefficient is about 3 × 10^–5^, three times greater than that observed for this water-soluble phase at 0% RH. Consequently, our direct measurements of reactive uptake demonstrate that multiphase processing of the water-soluble phase of BBOA is highly dependent on RH. From direct measurements of diffusion, it is known that the water-soluble (i.e., hydrophilic) phase of BBOA is plasticized by particulate water; for example, the diffusion coefficient of a fluorescing dye in hydrophilic BBOA, generated from pine, increased from roughly 1 × 10^–12^ cm^2^ s^–1^ at 0% RH to 1 × 10^–9^ cm^2^ s^–1^ at 75% RH, i.e., about 3 orders of magnitude, and eventually reached the diffusion coefficient of the dye in water, about 1 × 10^–6^ cm^2^ s^–1^ at saturation.^21^

We simulated the timeseries of uptake coefficient using KM-GAP. Four parameters were varied to fit to the experimental observations: the diffusion coefficient of ozone, the diffusion coefficient of BrC, the second-order rate constant between ozone and reactive BrC species, and the initial concentration of reactive BrC species. The diffusion coefficient of ozone at 75% RH was set to 8.9 × 10^–8^ cm^2^ s^–1^, in agreement with previous simulations for BBOA from pine.^21^ The rationale for this decision was that the viscosity of water-soluble BBOA, regardless of fuel species (i.e., whether pine or eastern red cedar) and combustion conditions, will approach that of water at saturation, so the diffusion coefficient at 75% RH will exhibit only slightly more variability with these factors. It is reasonable to expect larger differences due to these factors at 0% RH. Indeed, the optimized diffusion coefficient of ozone in this water-soluble phase at 0% RH was found to be 1.8 × 10^–8^ cm^2^ s^–1^, more than an order of magnitude higher than the value used for BBOA from pine, 1.1 × 10^–9^ cm^2^ s^–1^. The optimized diffusion coefficient of BrC from the model was 1.0 × 10^–10^ cm^2^ s^–1^ at 0% RH and 3.3 × 10^–9^ cm^2^ s^–1^ at 75% RH. Note that, for all experiments with nonirradiated BBOA, the number concentration of reactive BrC species was 3.6 × 10^17^ molecules cm^–3^, and the rate constant was 5 × 10^–17^ cm^3^ molecule^–1^ s^–1^. The value of the rate constant used here is within the range of previously measured rate constants between ozone and components representative of BrC. Rate constants between ozone and simple aromatics, such as toluene and xylenes, are in the range of 10^–21^-10^–19^ cm^3^ molecule^–1^ s^–1^, while rate constants between ozone and more complex aromatics and phenols are faster, in the range of 10^–17^-10^–15^ cm^3^ molecule^–1^ s^–1^ in water.^55−57^ Since phenols and other substituted aromatics are abundant in BBOA,^2^ our value was chosen to fall within this latter range. The simulated timeseries capture both the initial uptake and the slow decrease in the uptake over the 4-h exposure, as shown in Figure 3a.

It should be noted that the Henry’s law constant was kept the same at different RHs, but due to the hydrophobic nature of ozone, it may be higher at lower RH when less water is in the films, as shown in Berkemeier et al. for ozone dissolution in shikimic acid.^58^ Using a higher Henry’s law constant for the 0% case would require the films to have a lower diffusivity than presented here in order to maintain agreement with the measured uptake coefficients. As the variability of the Henry’s law constant in this range of RH is unknown for biomass burning material, here we maintain the same Henry’s law constant at different RHs to highlight that the differences in measured uptake coefficient can be explained by a higher viscosity at lower RH even in the absence of differences in solubility. Additionally, the increased uptake in the water-insoluble phase at 0% RH compared to the water-soluble phase could be due to increased solubility of ozone in the water-insoluble phase. Without additional constraints we cannot rule this possibility out. However, uptake into the water-insoluble phase at 75% RH was lower than uptake into the water-soluble phase at 75%, suggesting that mass transfer limitations are stronger in the water-insoluble phase than in the water-soluble phase at higher RH.

Additionally, the rate coefficient of ozone and reactive BrC was fixed for all films. While the value chosen is within the range of measured values for ozone oxidation of species representative of BrC in biomass burning samples, the actual value and variation between different films is unknown. While a variation in reaction rate coefficient at different RHs is not expected, a difference in reactivity could contribute to the variation in uptake coefficient observed for different solubility fractions and in the irradiated and nonirradiated cases. As the scraping of the films validated a variation in viscosity and there is no evidence for a reactivity variation, only a variation in diffusivity was simulated. Given the wide variety of species present in biomass burning, we note that a variation in either the reaction rate coefficient of the amount of reactive BrC in each film (taken together, being the reactivity) cannot be ruled out.

We next investigated reactive uptake of ozone onto the water-insoluble (i.e., hydrophobic) phase of BBOA. At 0% RH, reactive uptake onto this phase was greater than that onto the water-soluble phase, dropping by about 50%. The associated uptake coefficient is just below 1.5 × 10^–5^, similar to the value previously observed for whole BBOA at the same RH. From the multiphase kinetics model, the optimized diffusion coefficients for ozone and BrC were 2.7 × 10^–8^ cm^2^ s^–1^ and 8.0 × 10^–10^ cm^2^ s^–1^, respectively. These values are greater than those for the hydrophilic phase at 0% RH, indicating that the hydrophobic phase is less viscous than the hydrophilic phase at 0% RH. At 75% RH, the reactive uptake increased only slightly, such that the relative ozone mixing ratio was still about 50%, and the uptake coefficient reached 1.5 × 10^–5^. The optimized diffusion coefficient of BrC from the model at 75% RH was 9.0 × 10^–10^ cm^2^ s^–1^, 13% higher than the value at 0% RH. This weak response to RH is consistent with the composition of this phase; i.e., since these components are not soluble in water, they do not readily take up water and are not plasticized. Furthermore, this weak response is consistent with direct measurements of the diffusion coefficient of dye in hydrophobic BBOA from pine, which gave a value on the order of 1 × 10^–11^ cm^2^ s^–1^ that was steady until RH approached saturation;^21^ interestingly, the magnitude of this diffusion coefficient lies between the values measured for the hydrophobic phase at 0 and 75% RH, respectively. Our direct observations of reactivity show that the multiphase processing of the hydrophilic phase of BBOA at low and high RH indeed bookends that of the hydrophobic phase across the same RH range. Consequently, despite differences in absolute diffusion coefficients, this trend is shared between BBOA generated from different species and experimental setups.

The optimized diffusion coefficients for these nonirradiated BBOA components are broadly consistent with qualitative microscopy experiments. In these experiments, thin films alternately prepared on flat, circular cover glasses were scraped and observed 0, 2, and 20 h afterward. Results for a thin film of the water-insoluble components of nonirradiated BBOA at 35% RH and 298 K are shown in Figure S3. At 0 h, the channel, less than 50 μm across, was rectangular, with sharp edges with some irregular features. After 2 h, the roughness of the edges was decreased. After 20 h, the edges were smooth and rounded, and the channel width had decreased slightly, consistent with the flow of a viscous liquid. All of the optimized diffusion coefficients of BrC discussed above are greater than 10^–11^ cm^2^ s^–1^, which corresponds to a viscosity of 10^3^ Pa s, e.g., similar to that of peanut butter.^59^

### Effects of Irradiation

Motivated by our previous observations of significantly reduced reactive uptake to thin films of whole BBOA after irradiation,^26^ we next investigated the effects of irradiation on the separated water-soluble and insoluble phases at 0% RH, as shown in Figures 2b and 3b. We note that these experiments probe multiphase chemistry after irradiation, rather than during illumination, an approach that has been used by others in the past to demonstrate photoenhanced ozone uptake, which may also be an important pathway in the atmosphere.^60,61^ For the water-insoluble phase, the ozone mixing ratio initially decreased by 20% (see Figure 2b), associated with an uptake coefficient of about 5 × 10^–6^. This uptake coefficient is about one-third of that observed for the same phase and RH without irradiation. The ozone mixing ratio recovered quickly to a steady state, associated with an uptake coefficient of 5 × 10^–7^ (see Figure 3b), at least an order of magnitude lower than that observed without irradiation (see Figure 3a). To represent this low steady-state uptake in the simulations, the KM-GAP model predicted a diffusion coefficient of ozone of 3.5 × 10^–9^ cm^2^ s^–1^, lower than any of the values above. The diffusion coefficient of BrC was set to 1.4 × 10^–15^ cm^2^ s^–1^, supported by the qualitative microscopy experiments. After scraping the thin film made from irradiated water-insoluble components, a jagged groove that is wider than 50 μm was observed, as shown in Figure S3. The initial contents of the groove were shards, displaced to the edges of the groove, which are also jagged with many sharp edges. After allowing 2 and 20 h for equilibration, we observed no smoothing of these jagged features, in either the groove or the small shards, indicating a lack of flow consistent with a solid. At this low magnitude, the diffusion coefficient of BrC no longer affects the shape of the timeseries, so it cannot be further constrained. For the water-soluble phase, even less reactive uptake was observed after irradiation, with an uptake coefficient below 1 × 10^–6^ for the duration of the experiment. Previously, we similarly observed a significant decrease in reactive uptake of ozone onto whole BBOA after UV irradiation, and we attributed the decrease to increased viscosity due to oligomerization, supported by mass spectrometry and volatility tandem differential mobility analysis.^26^ There have been previous observations of oligomerization through light-driven processing, e.g., photosensitized reactions of pyruvic and oxalic acids.^62^ Here, the KM-GAP modeling and qualitative microscopy experiments further support that UV irradiation of whole and liquid–liquid phase-separated BBOA can significantly increase viscosity. To capture the shape of the experimental timeseries, i.e., the rapid approach to steady-state, for the hydrophobic phase after irradiation, the concentration of reactive BrC was decreased to 4.4 × 10^16^ molecules cm^–3^. This decrease in the concentration may be consistent with oligomerization; if the size of molecules at the surface increases, potentially the number of reactive moieties at the surface decreases.

Irradiation also resulted in changes to the light absorption of the water-soluble and insoluble phases (see Figure S1). After irradiation, the MAC values for the water-soluble BBOA at 365 and 405 nm were 0.19 ± 0.02 m^2^ g^–1^ and 0.11 ± 0.02 m^2^ g^–1^, respectively; those for the water-insoluble phase were 0.65 ± 0.01 m^2^ g^–1^ and 0.24 ± 0.02 m^2^ g^–1^ at the same wavelengths. These values are all lower than the corresponding values before irradiation, discussed above. At 405 nm, irradiation led to a loss of about 30% and 40% of the initial absorptivity for the water-soluble and insoluble phases, respectively. Interestingly, this whitening contrasts with the effect of the same period of irradiation on whole BBOA, prepared similarly to the individual phases here; i.e., for whole BBOA, irradiation led to an absorption enhancement of 40% at 405 nm.^26^ Light-driven processing involves in part the photolysis of carbonyl compounds, which are abundant in BBOA,^2,3^ through Norrish Type I reactions,^63,64^ followed by radical–radical recombination, leading to large, highly functionalized products.^26,65^ These products contribute to the increased viscosity discussed above. Broadly, functionalization and oligomerization are associated with increased light absorption.^18,66^ Light-driven processing of BBOA has been shown to also involve energy transfer from organic triplet excited states, ^3^C*, to dissolved molecular oxygen, leading to excited singlet oxygen, ^1^O_2_.^67−69^ Other reactive oxygen species, including OH radicals and superoxide, can be generated via photosensitized reactions,^68^ which in turn can initiate oxidation. The above discrepancy suggests that the relative roles of these processes change with the composition of the BBOA fraction. Indeed, differing evolution in light absorption has been observed for mixed and individual BBOA constituents that are photosensitizers.^70,71^ Future work should further explore to what extent and under what conditions the effects of irradiation on viscosity and light absorption are coupled in whole and phase-separated BBOA.

### Atmospheric Implications

We further applied KM-GAP to estimate the lifetime of the original, primary molecules in the water-soluble and insoluble phases of BBOA with respect to multiphase ozone oxidation. The above simulations for thin films allow us to implement the derived parameters for aerosol particles, which have increased surface area-to-volume ratios and may exhibit enhanced uptake.^72^ It is important to qualify that our approach here is focused on initial constituents that react with ozone, and we assume that every molecule of ozone lost to reactive uptake consumes one molecule of the primary BBOA components. Since first-generation products of ozone oxidation may still be light-absorbing, these lifetimes do not reflect the lifetime of light absorption, nor do they account for other aging mechanisms, like multiphase hydroxyl and nitrate radical-initiated oxidation;^14,16^ nonetheless, these lifetimes importantly allow new insights into the reactivity of primary BBOA components in different phases upon LLPS.^21^ It should also be noted that the lifetime values presented here are highly dependent on the best fit parameters in Table S2. Given the uncertainty in those parameters, the lifetime values will be used for general comparisons between cases, but should not be interpreted as specific lifetimes of reactive BrC in the atmosphere. Given that the experimental study focuses on macroscopic films in a flow tube, the extension to atmospheric aerosol particles highlights how the results fit into the relevant atmospheric context.

**Figure 4:**
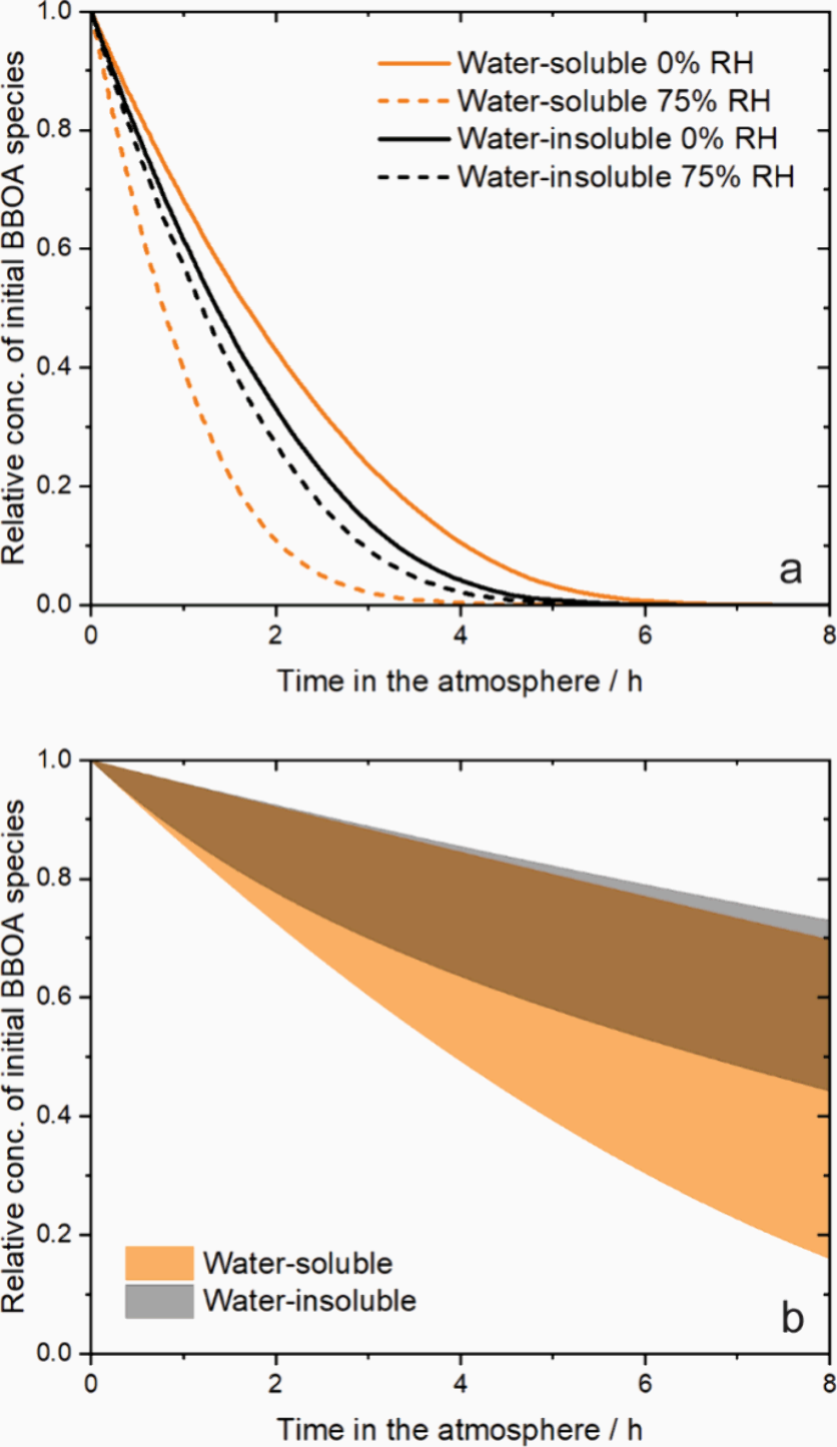
Modeled decay of initial BBOA species in the atmosphere due to multiphase ozone oxidation (a) before and (b) after UV irradiation at 293 K and an ozone mixing ratio of 35 ppb. In the bottom panel, the RH is 0%. In panel b, the shaded regions are semitransparent, so the darkest region in the middle is where the two conditions overlap.

The estimated decay in initial BBOA components for a 300 nm particle suspended in the atmosphere at 293 K and an ozone mixing ratio of 35 ppb is illustrated in Figure 4. Among nonirradiated BBOA, the decay is fastest for the water-soluble phase at high RH, with an associated lifetime of 1.1 h, and slowest for the same phase at low RH, with an associated lifetime of 2.4 h. The decay of BBOA in the water-insoluble phase is nearly the same at 0 and 75% RH, in keeping with the experimental uptake coefficients, associated with lifetimes of 1.9 and 1.6 h, respectively. These lifetimes are shorter than those previously modeled for BBOA from pine,^21^ but the trend between the phases and RH values is consistent, as discussed above. Consequently, LLPS of BBOA, resulting in a core of water-soluble BrC inside a shell of water-insoluble BrC, could restrict reactivity at moderate to high RH, typical of ambient conditions,^21^ at which the shell of water-insoluble components would shield the core from reactions with ozone. This restriction may be expected to increase as temperature decreases at higher altitudes as the shell phase would become more viscous.^21,73^

The modeled decay due to multiphase ozone oxidation is highly dependent on the mixing ratio of gas-phase ozone, which varies widely in biomass burning plumes. From field measurements during the BORTAS campaign, for example, the distribution of ozone mixing ratios exhibits appreciable relative frequencies from about 20 to 80 ppb; the distribution is bimodal with peaks centered at about 30 and 50 ppb.^74^ As shown in Figure S4, at the lowest mixing ratios observed in plumes, i.e., 20 ppb, the lifetimes are longer and distributed across a wider absolute range, e.g., about 2 and 4 h for BrC in the water-soluble phase at 0 and 75% RH, respectively. At 50 ppb, the lifetimes are all shorter than 2 h, but the lifetime in the water-soluble phase at 0% RH is still about twice as long as that at 75% RH. We note that, since the estimated decay is based on the loss of ozone from the gas-phase, rather than changes in composition or absorption of the condensed phase, it reflects the consumption of primary BrC chromophores. The products of ozone oxidation of aromatic rings or exocyclic double bonds, e.g., in cinnamaldehydes, may still absorb visible light to some degree. Although whitening may be expected eventually for BBOA undergoing ozone oxidation, as has been reported in the past,^12,13,75^ we did not regularly quantify optical properties after ozone exposure. By design, these experiments focus on the gas phase (i.e., loss of ozone) rather than the condensed phase. We note that the thin films are still macroscopic samples, so the reservoir of BBOA molecules is much larger than the number of reactive collisions over the course of a 4-h exposure, so changes in the bulk properties were not expected.

The significant increase in viscosity upon irradiation of both the water-soluble and insoluble phases results in much slower decay due to multiphase ozone oxidation after the BBOA has been exposed to UV radiation. The ranges of estimated decay represent the uncertainty carried through from the optimized parameters. For BrC in the water-soluble phase, the range of lifetimes is about 18–26 h at an ozone mixing ratio of 35 ppb; in the water-insoluble phase, the range is about 5–10 h, consistent with the greater (although still small) uptake observed for the water-insoluble phase in the flow tube after irradiation. As above, these lifetimes decrease with increasing ozone mixing ratio (see Figure S4). Our experimental and modeled results further support the significant impact of UV irradiation on the viscosity of BBOA,^26^ analogous to the impact observed for secondary organic aerosol from biogenic precursors.^65^

The relative roles of oxidation and irradiation will be important in dictating the fate and impact of the water-soluble and insoluble phases of BBOA in the atmosphere. While the effects of RH on the multiphase processing of the water-soluble phase are evident in the variable reactive uptake and estimated lifetime, both phases are viscous liquids across the wide range of RH explored here. In the future, experiments at lower temperatures, relevant to the upper troposphere, should be performed to determine to what extent the phases are vitrified there. Also, the estimated lifetimes with respect to multiphase ozone oxidation are shorter than the equivalent residence time in the atmosphere associated with the experimental UV irradiation, i.e., 3 d. Again, these lifetimes reflect the consumption of primary BrC chromophores. Even if products were fragments and no longer absorbed at visible wavelengths, they would still absorb at UV wavelengths, which could drive oligomerization. A limitation of the current data set is that it does not include samples that were irradiated before and after significant oxidation. In the future, oxidation and irradiation should be explored in alternate sequences to determine whether the effects of these processes on the composition, absorptivity, and reactivity of the polarity fractions of BBOA are commutative. Altogether, our current results suggest that LLPS and UV irradiation both restrict the reactivity of BrC in BBOA particles in the atmosphere, potentially prolonging its positive radiative forcing and, in turn, warming effect on climate.

## Supporting Information Available

Figures of UV–vis spectra in terms of MAC, timeseries of reactive uptake with standard deviation, and estimated lifetimes as a function of ozone mixing ratio; photographs of microscopy experiments on thin films of nonirradiated and irradiated water-insoluble BBOA; tables of physical properties and parameters for coated-wall flow-tube experiments and best-fit parameters from the KM-GAP simulations. A previous version of this manuscript appeared as a preprint (20.500.14446/344834) (PDF)
