# Supplementary material for: Multiphase Processing of the Water-Soluble and Insoluble Phases of Biomass Burning Organic Aerosol
Source: ACS EST Air. 2025 Mar 31;2(4):637–47. doi: 10.1021/acsestair.4c00345 (PMC11997950; doi:10.1021/acsestair.4c00345)
Supplement: Supplementary file 1 — ea4c00345_si_001.pdf [file ea4c00345_si_001.pdf]

Supporting Information for

Multiphase Processing of the Water-Soluble and Insoluble Phases of Biomass Burning Organic Aerosol

Habeeb H. Al-Mashala[1], Meredith Schervish[2], Sithumi M. Liyanage[1], Jace A. Barton[1], Manabu Shiraiwa[2], and Elijah G. Schnitzler[1,]*

[1] Department of Chemistry, Oklahoma State University, Stillwater, OK 74078, USA

[2] Department of Chemistry, University of California Irvine, Irvine, CA 92697, USA

E-mail: elijah.schnitzler@okstate.edu.

Contents

Figures S1-S4

Tables S1-S2

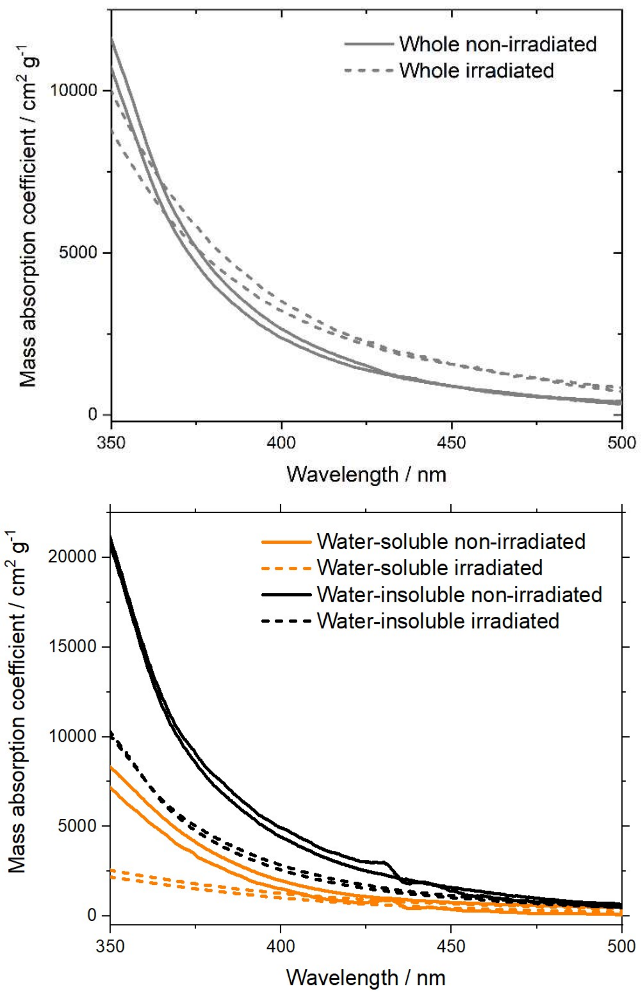

Figure S1. Absorption in terms of mass absorption coefficients for (top) whole BBOA and (bottom) water-soluble and insoluble phases of BBOA before and after UV irradiation. Two curves are shown for each condition, representing replicate treatments on different samples. The curves for whole BBOA are taken from Ref. 26.

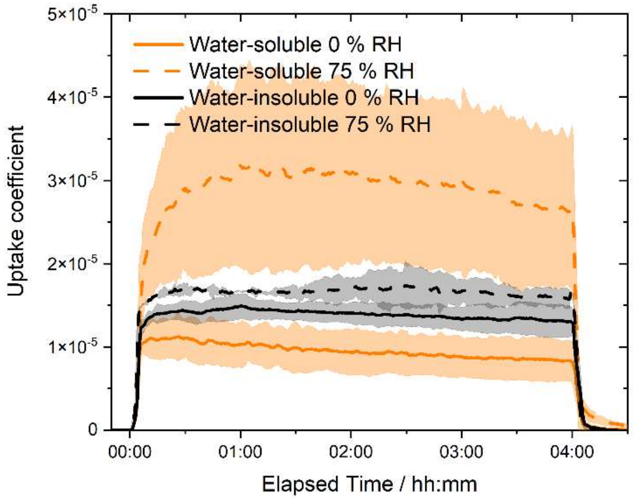

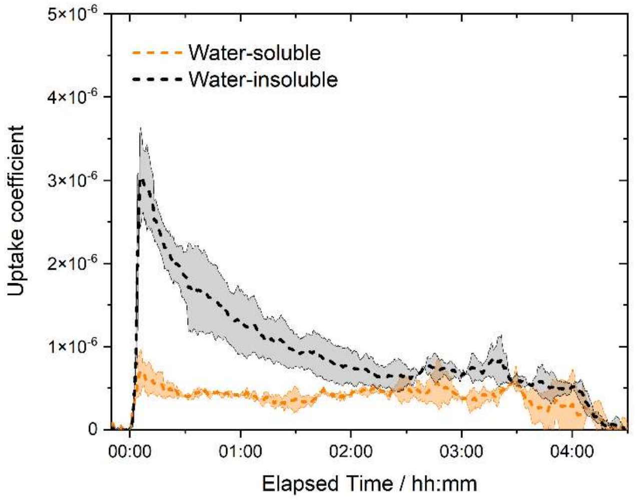

Figure S2. Experimental uptake coefficient as a function of elapsed time for (top) non-irradiated and (bottom) irradiated BBOA. In the bottom panel, the RH is 0%. The shaded regions indicate the standard deviation from repeated experiments.

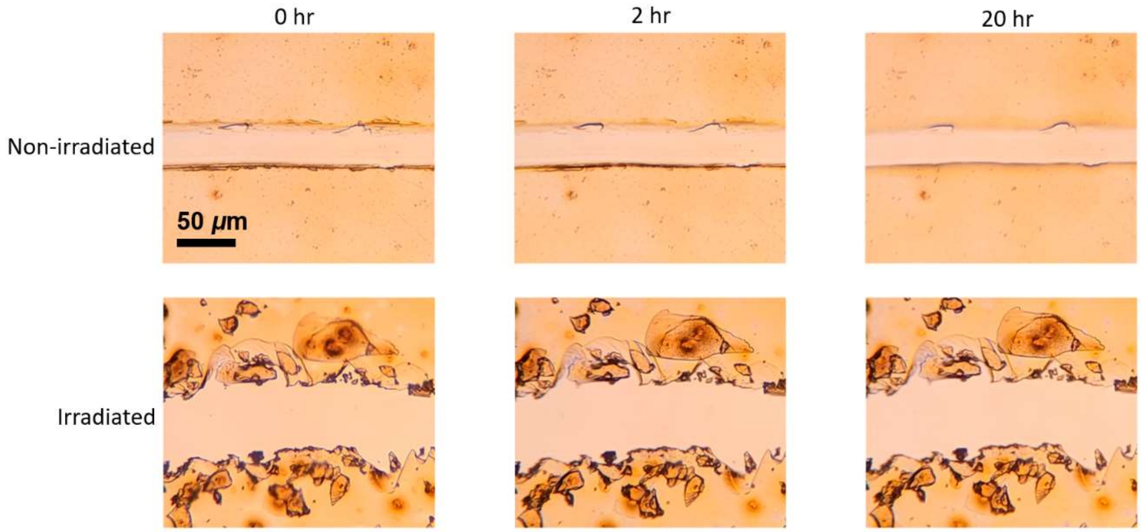

Figure S3. Micrographs of thin films of non-irradiated and irradiated water-insoluble BBOA alternately prepared on flat glass substrates at intervals of time after scraping.

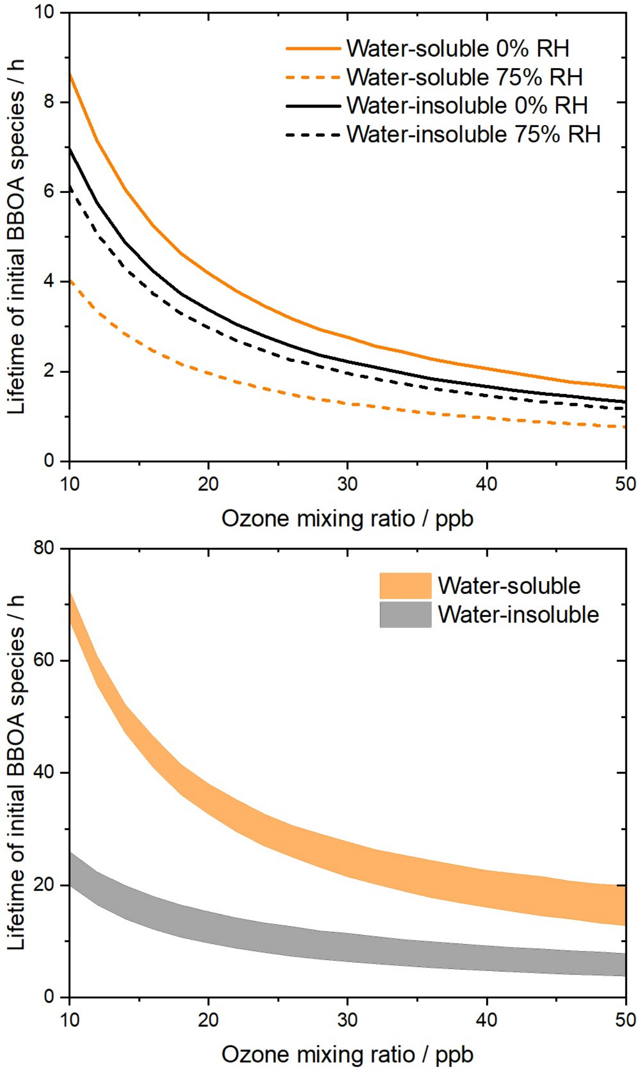

Figure S4. Estimated lifetimes of initial BBOA species in the atmosphere with respect to multiphase ozonolysis as a function of ozone mixing ratio (top) before and (bottom) after UV irradiation. In the bottom panel, the RH is 0%.

Table S1. Parameters for the coated-wall flow-tube setup.

Parameter (symbol)	Value	Formula	Units
Temperature (T)	296.5		K
Pressure (P)	1		atm
Flow tube diameter (D_{tube})	2.45		cm
Flow tube cross-sectional area (A)	4.71	$A = \pi r^2$	cm^2
Volumetric flow rate (F)	0.30		L min^{-1}
Linear velocity (v)	1.06	$v = F/A$	cm s^{-1}
Length of coated tube (L)	5		cm
Residence time (t)	4.71	$t = L/v$	s
Reynolds number (Re)	17.3	$\mathrm{Re} = (\rho * D_{tube} * v)/\eta$	
Length to laminar flow (l)	1.48	$l = 0.035 * \mathrm{Re} * D_{tube}$	cm
Mean molecular velocity (ω)	362	$\omega = \mathrm{sqrt}(8RT/(\pi M))$	m s^{-1}
Ozone diffusion coefficient (D)	0.13		cm^2 s^{-1}
Mean free path (λ)	1.08E-05	$\lambda = 3D/\omega$	cm
Density of air (ρ)	1.2		kg m^{-3}
Viscosity of air (η)	1.80E-05		Pa s
Knudsen number (Kn)	8.81E-06	$\mathrm{Kn} = 2\lambda/D_{tube}$	
Dimensionless axial distance (z^*)	0.204	$z^* = z(\pi D/2F)$	
Sherwood number (N_{Shw})	4.10	$3.6568 + A/(z^* + B)$	

Table S2. Best-fit parameters from the KM-GAP simulations.

Condition	$D_{b,O3}$ / $cm^2\ s^{-1}$ (this work)	$D_{b,O3}$ / $cm^2\ s^{-1}$ (Gregson et al.[a])	$D_{b,BrC}$ / $cm^2\ s^{-1}$ (this work)	$D_{b,BrC}$ / $cm^2\ s^{-1}$ (Gregson et al.[a])	k_{BR} / $cm^3\ s^{-1}$	[BrC] / cm^{-3}
WS 0%	1.75e-8	1.067e-9	1e-10	7.028e-12	5e-17	3.6e17
WS 75%	8.905e-8	8.905e-8	3.295e-9	4.521e-9	5e-17	3.6e17
WIS 0%	2.7e-8	3.083e-9	8e-10	3.31e-11	5e-17	3.6e17
WIS 75%	3.5e-8	3.295e-9	9e-10	3.65e-11	5e-17	3.6e17
WS Irr 0%	5.5e-10	N/A	1.39e-15[b]	N/A	5e-17	4.37e16
WIS Irr 0%	3.5e-9	N/A	1.39e-15[b]	N/A	5e-17	4.37e16

[a] Comparison to values in Ref. 21.

[b] Does not impact the fit in this range and therefore cannot be constrained.
